# Irish farmers’ interactions with regional veterinary laboratories- reasons, results, reactions: a survey

**DOI:** 10.1186/s13620-022-00225-6

**Published:** 2022-09-27

**Authors:** Aideen Kennedy, Ian Hogan, Rebecca Froehlich, Shane McGettrick, Cosme Sánchez-Miguel, Micheál Casey, Maresa Sheehan

**Affiliations:** 1 Kilkenny Regional Veterinary Laboratory, Department of Agriculture, Food and the Marine, Kilkenny, Ireland; 2 Limerick Regional Veterinary Laboratory, Department of Agriculture, Food and the Marine, Limerick, Ireland; 3 Sligo Regional Veterinary Laboratory, Department of Agriculture, Food and the Marine, Sligo, Ireland; 4 Cork Regional Veterinary Laboratory, Department of Agriculture, Food and the Marine, Cork, Ireland; 5 Regional Veterinary Laboratories Division, Backweston Campus, Department Agriculture, Food and the Marine, Celbridge, Kildare, Ireland

**Keywords:** Animal health, Disease surveillance, Laboratory submissions, Post-mortem examination

## Abstract

**Background:**

Animal health surveillance is important in ensuring optimal animal health and welfare. Monitoring of diagnostic submissions, including post-mortem examination of carcasses, at the Department of Agriculture Food and the Marine laboratories, provides the basis for this type of passive surveillance in Ireland. The process requires engagement from veterinarians and farmers from all sectors of the agricultural spectrum. This study aims to identify the reasons why farmers engaged in dairy, beef, sheep, and mixed farming enterprises submit carcasses or not to the Regional Veterinary Laboratories.

**Results:**

Surveys were distributed in hard copy format at Regional Veterinary Laboratories, and fifty Teagasc facilitated farmer discussion groups. There were 1179 responses collected in 54 locations. The top reasons participants submitted to the laboratories were 1) to guide treatment/ vaccination, 2) fear of a contagious disease, and 3) if their veterinarian advised them to. The top reasons for not submitting were 1) the vet making a diagnosis on the farm, 2) the distance from the laboratory, and 3) lack of time and labour. Implementation of vaccination protocols was the main change implemented based on results, followed by management changes and the use of different treatments, e.g., switching from antibiotic to parasite treatment. Sheep enterprises were more likely than dairy to choose distance and cost as a reason not to submit. Dairying enterprises were more likely than other enterprise types to submit if they feared a contagious or zoonotic disease.

**Conclusion:**

Positively, this survey shows the desire of participants to submit to the laboratories to guide treatment and vaccination protocols, potentially indicating that positive engagement between stakeholders and the RVLs will help promote optimal animal health and promote responsible antimicrobial use. Results also show the critical role of veterinarians in continued disease surveillance on farms. Maintaining engagement with all farming sectors will be essential in promoting successful animal health surveillance.

## Background

Animal health surveillance is important in ensuring optimal animal health and welfare. In turn, this is required to protect public health and allow access to international markets. Department of Agriculture Food and the Marine (DAFM) central and Regional Veterinary Laboratories (RVLs), situated across six locations in the Republic of Ireland, play an essential role in national animal disease surveillance in the Republic of Ireland. This includes monitoring trends in animal health resulting from new, re-emerging, endemic and exotic diseases. Enhanced passive surveillance encourages producers to report disease with active follow-up of suspect disease reports [1]. Monitoring of diagnostic submissions by private veterinary practitioners (PVPs) to DAFM laboratories provides the basis for this type of surveillance in the Republic of Ireland; the degree of participation of PVPs and farmers hugely affects disease reporting rates. *Post-mortem* examinations (PME) performed on carcasses referred by PVPs are of particular importance in early warning surveillance and when investigating trends in endemic diseases, as PME offers the opportunity for optimum sample matrix selection, the possibility of further laboratory assessment and allows sample storage, accessible for future reference. DAFM provides a partially subsidised PME service throughout the RVL network. Results are issued to the farmers’ own PVP, with PVPs responsible for relaying results back to the farmer and assisting with result interpretation and implementation of measures. A carcass cannot be submitted to an RVL without veterinarian referral; therefore, the process requires engagement from both PVPs and farmers. The service aims to be of mutual benefit to the farmers/ vets who receive information regarding cause of death, the data generated aids DAFM with surveillance information.

Examination of factors influencing laboratory submission have previously been reported from veterinarian’s perspective [2–4]. A recent study also examined factors involved in dairy farmers decision to submit to DAFM laboratories [5]. The National Farmed Animal Biosecurity Strategy [6] indicated the requirement to identify knowledge gaps in relation to factors constraining and promoting the adoption of good biosecurity practices in Ireland, and DAFM Animal Health Surveillance Strategy [7] indicated the importance of engagement from all stakeholders, and as such this study aims to identify factors involved in sample/ carcass submission to the RVLs by participants engaged in dairy, beef, sheep and mixed farming enterprises.

## Materials and methods

Questions were compiled based on information gathered from peer reviewed publications and DAFM research officer, veterinarian, and farmer experience of submission to RVLs. The study was piloted amongst a small number of farmers and following minor revisions was distributed in hard copy format at regional veterinary laboratories and fifty Teagasc (Irish Agriculture and Food Development Authority) facilitated farmer discussion groups located across the Republic of Ireland. Survey participation was voluntary, and consent was sought prior to partaking. The survey was non – incentivised and anonymous.

### Descriptive analysis and herd classification

Hardcopy survey responses were entered into an online survey software package (www.surveymonkey.com) with electronic inputs being manually checked against hardcopy versions. Coded responses to each question were subsequently downloaded and Microsoft Excel (MS Office, Version 2010) used to organise the data, and complete descriptive analysis. Enterprise type was categorised into dairy only, mixed (dairy and beef stock), beef only, sheep only, sheep mixed (with dairy or beef) and those with young dairy stock only aged less than 1 year. The median stock number on the farms of participants was attained (123 animals), and the stock number was categorised into below-median stock number and above-median stock number.

### Statistical analysis

Statistical analysis, namely logistic regression, was completed using Stata data analysis and statistical software (Version 12). A manual backwards elimination with a forward step was applied to each model, with significant variables (*p* ≤ 0.05 chosen as accepted significance level) retained in the final model. Independent variables included in the models were enterprise type, herd owner or not, sex, age (categorised into < 40 years, 40–65 years and >  65 years), and above/below median stock number. Dependent variables included- whether participants submitted samples to the RVLs or not and reasons why or why not participants submitted samples to the RVLs. Respondents were asked to pick their top three reasons why they would/ would not submit; however, a number of respondents ranked all answer options from 1 to 12. Therefore for logistic regression analysis answers were categorised into being selected as a top 3 reason or not.

## Results

**Fig. 1:**
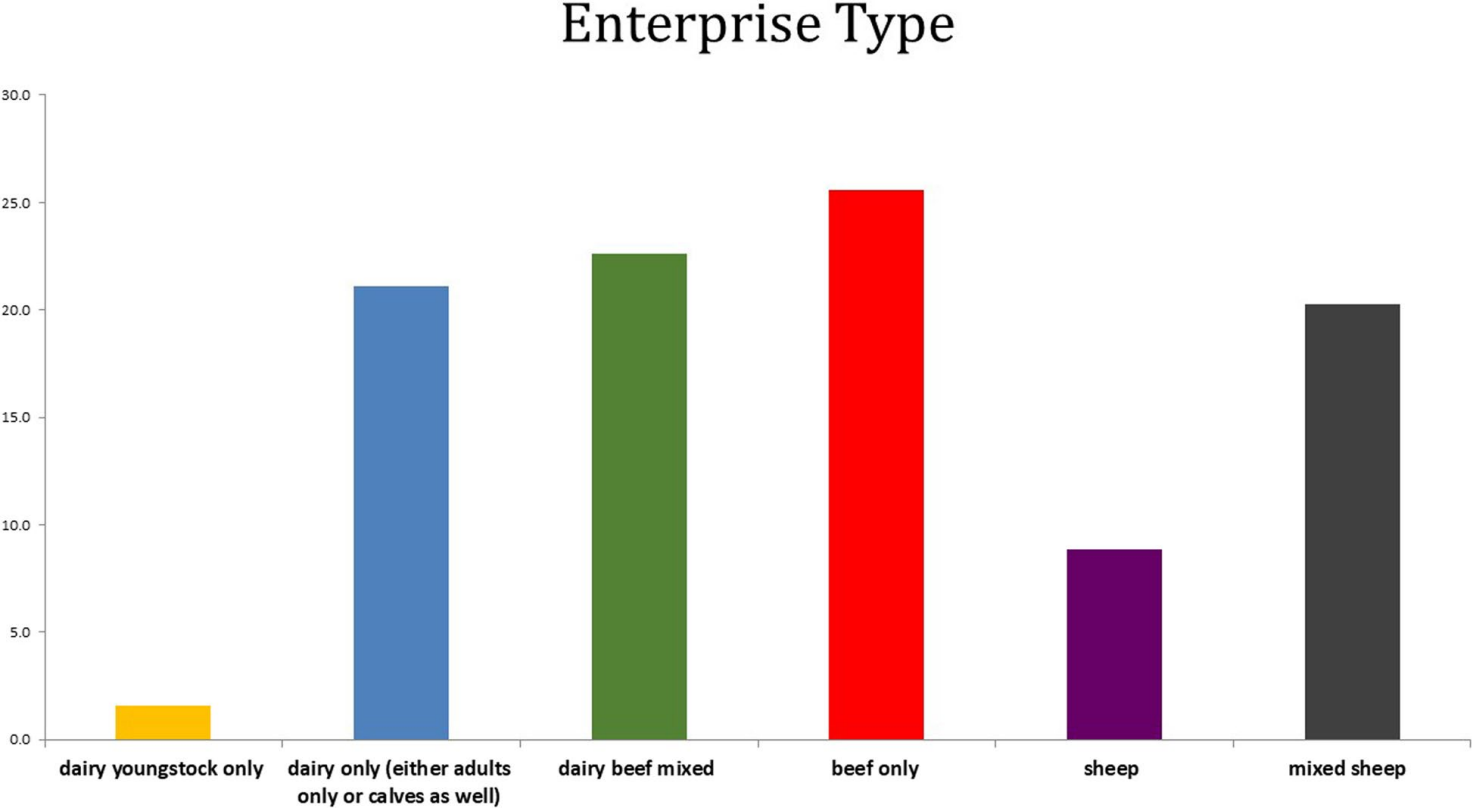
Enterprise type

**Fig. 2:**
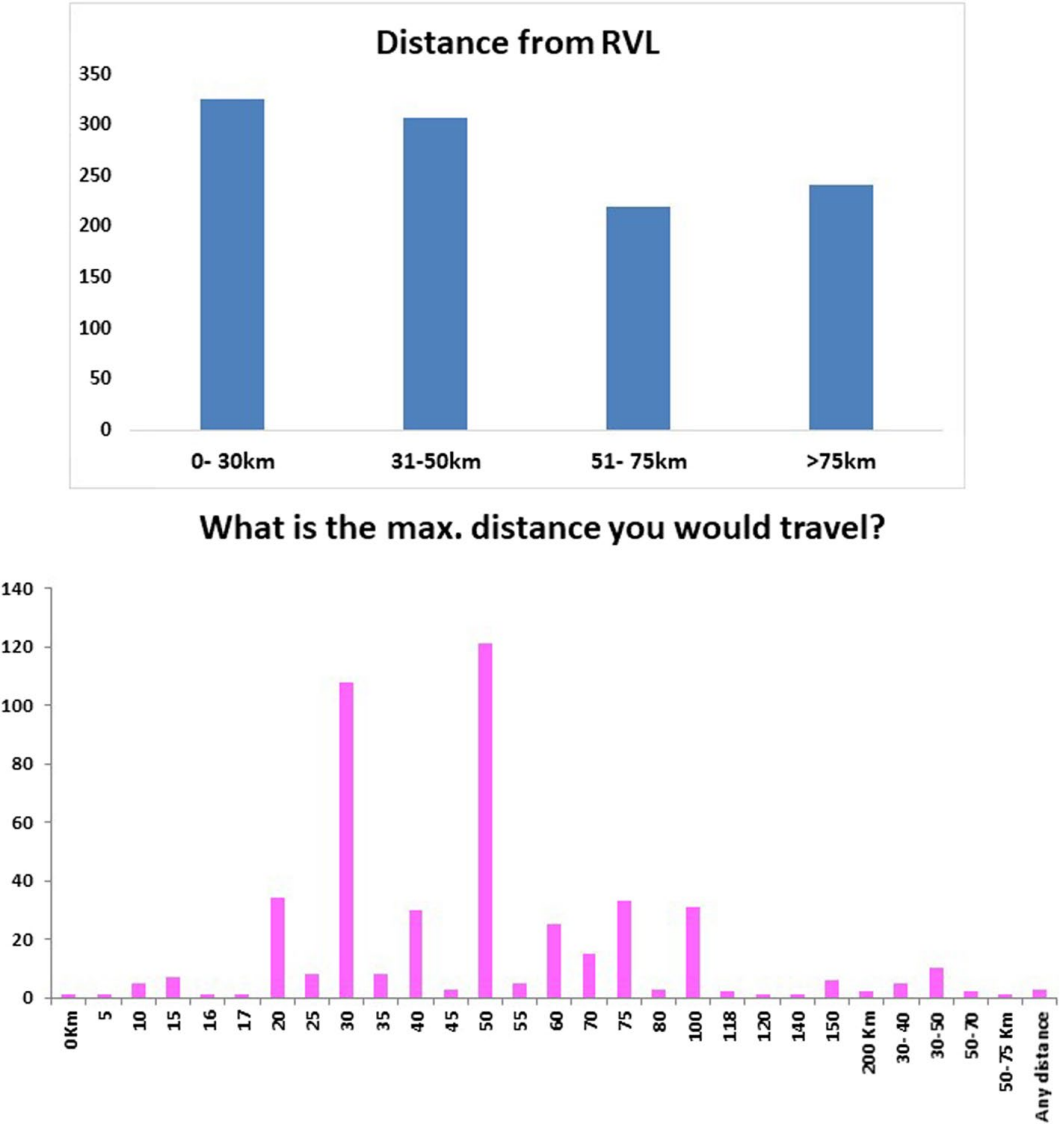
Distance from RVL a) current distance to RVL b) hypothetical maximum distance participants would travel to submit to RVL

**Fig. 3:**
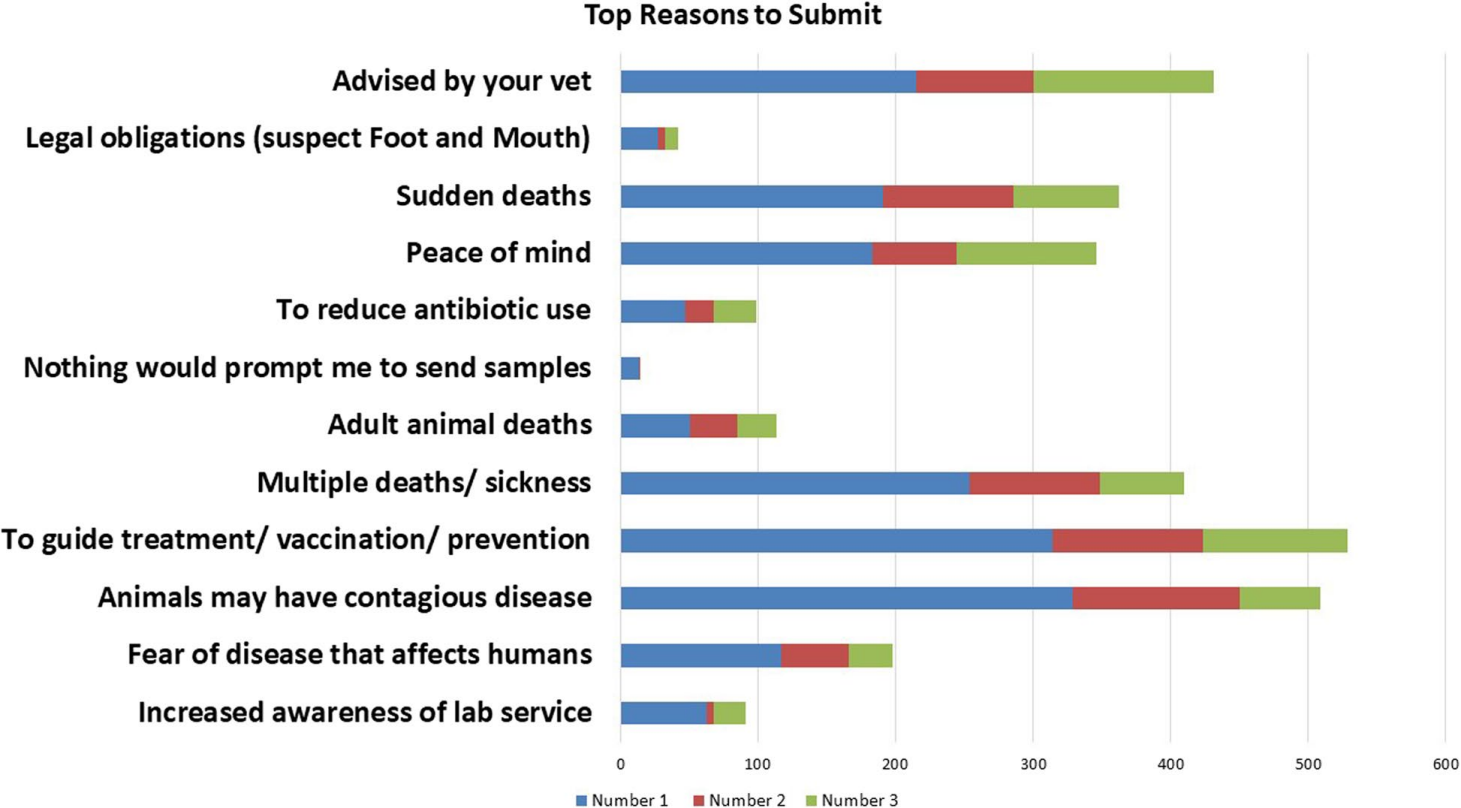
Top reasons to submit to the RVLs

There were 1179 responses collected in 54 locations. Four hundred twenty-nine of the surveys were collected at the RVLs, with the remainder collected at Teagasc discussion groups. Most respondents were aged between 40 and 65, 20 *%* were under 40, and 15 *%* were over 65. Respondents were predominantly the owner of the herd (95.9%). Over 70% of respondents classified themselves as full-time farmers. Less than 5% of those surveyed were female. Samples had previously been submitted to the RVLs by over 60% of those surveyed. Of those that submitted, over 25% had submitted within the previous 12 months. The highest number of those surveyed were beef only farmers (> 25%), followed by mixed enterprises and dairy only (22 and 21%, respectively) (Fig. 1). Most respondents estimated their distance to their nearest laboratory between 0 and 30 km (Fig. 2). However, when the results of those who never submitted to the RVL results were analysed independently, over 35% of non-submitters estimated a distance of over 75 km to their nearest laboratory. Some respondents picked two distance options, possibly indicating they submitted to more than one laboratory. When asked the hypothetical maximum distance they would travel to submit a carcass, the answers ranged between 0 km to 200 km. Two respondents answered that they would travel any distance required in order to submit. Sheep farmers had higher odds of selecting distance as a top reason not to submit to the RVL compared to dairy farmers. The top reasons participants would submit to the RVL were 1) to guide treatment/vaccination, 2) fear of a contagious disease, and 3) if their PVP advised them to (Fig. 3). When non-submitters were analysed on their own, the top reason they would submit would be if their PVP advised them.

**Fig. 4:**
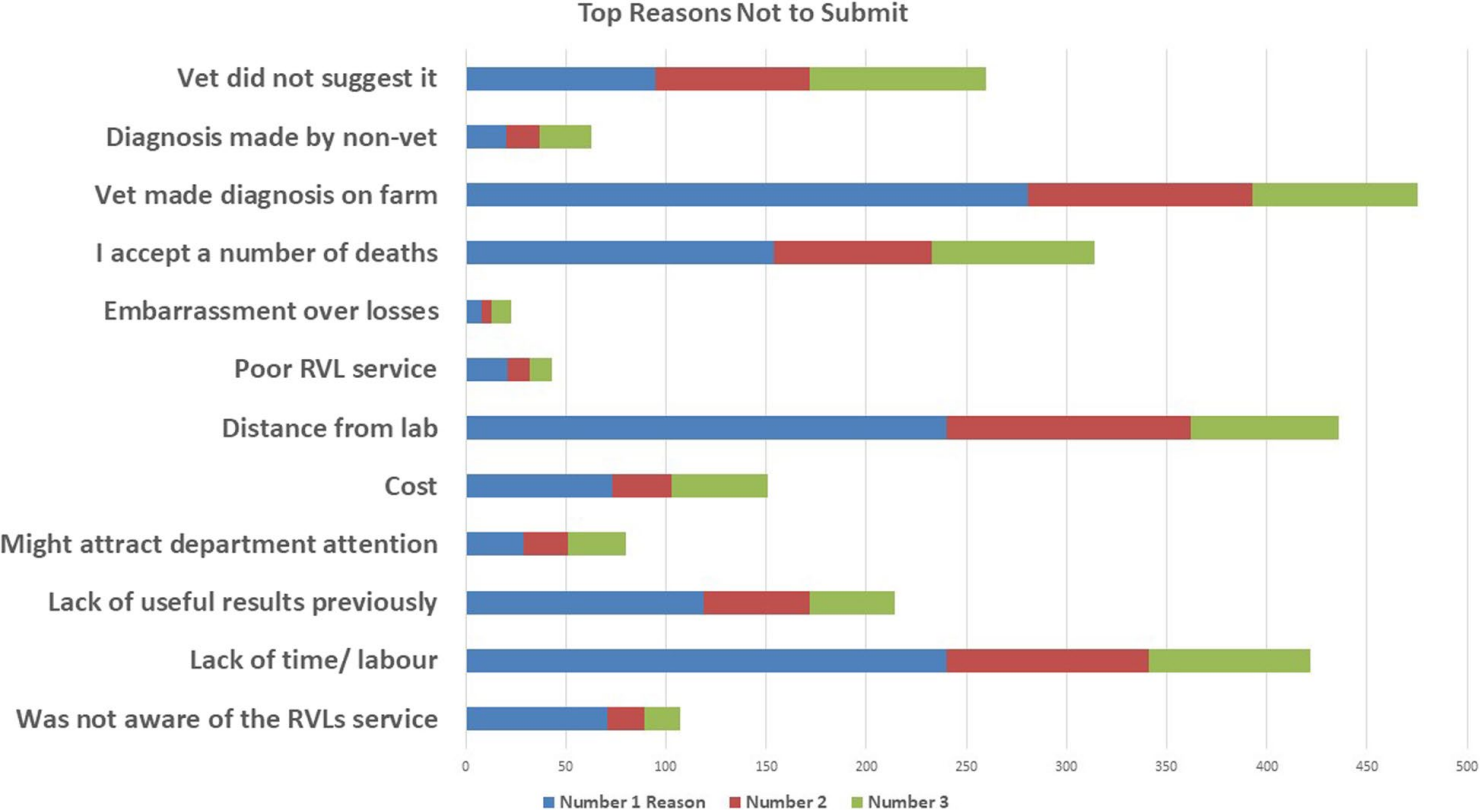
Top reasons not to submit samples to RVLs

**Fig. 5:**
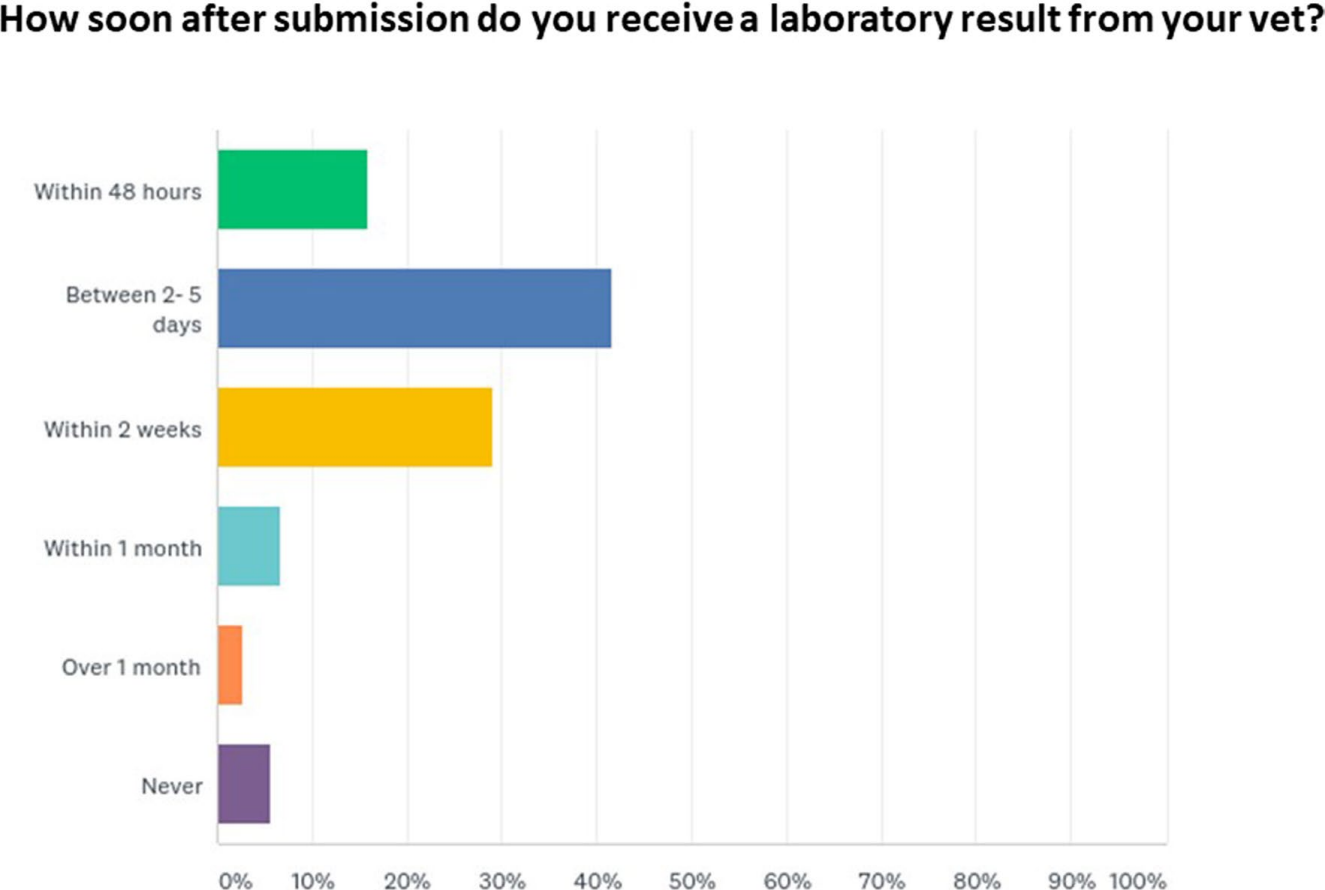
Speed participants received their results

When all responses were examined (both submitters and non-submitters), the top reasons for not submitting to the lab were 1) the vet making a diagnosis on the farm, 2) the distance from the lab or 3) lack of time and labour (Fig. 4). However, when responses of those who never submitted to the lab were examined on their own, the top reason was the distance from RVLs. A number of participants listed other reasons for not submitting; these included low mortality rates and not having any reason to submit, previous inconclusive results, slow receipt of results, never receiving results from their PVP (Fig. 5), advice being too general and scavenging of carcasses preventing them being submitted. More than half received their results verbally, 23.2% contacted their vet to receive results, with lesser numbers receiving results via email, post or text message.

**Fig. 6:**
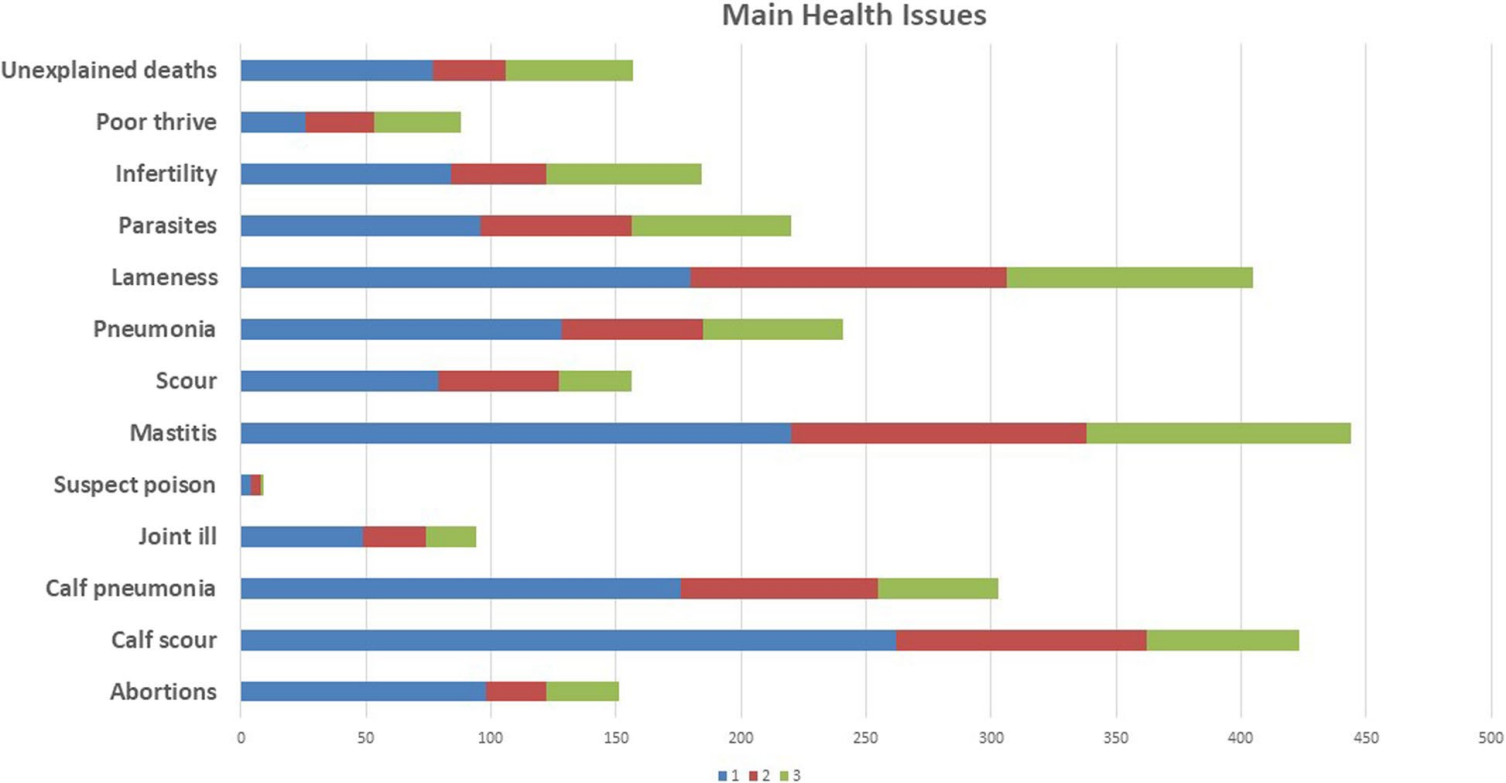
Main health issues identified on participants farm

The primary health issues identified were mastitis, lameness, calf health issues, parasites, and pneumonia (Fig. 6). In addition to the response options offered, some listed Johne’s disease, Tuberculosis, digital dermatitis, milk fever, summer scour syndrome, orf, *Mycoplasma bovis*, calving issues and redwater as problems on their farms. Bad weather and foxes (presumably taking lambs) were also noted by individuals.

**Fig. 7:**
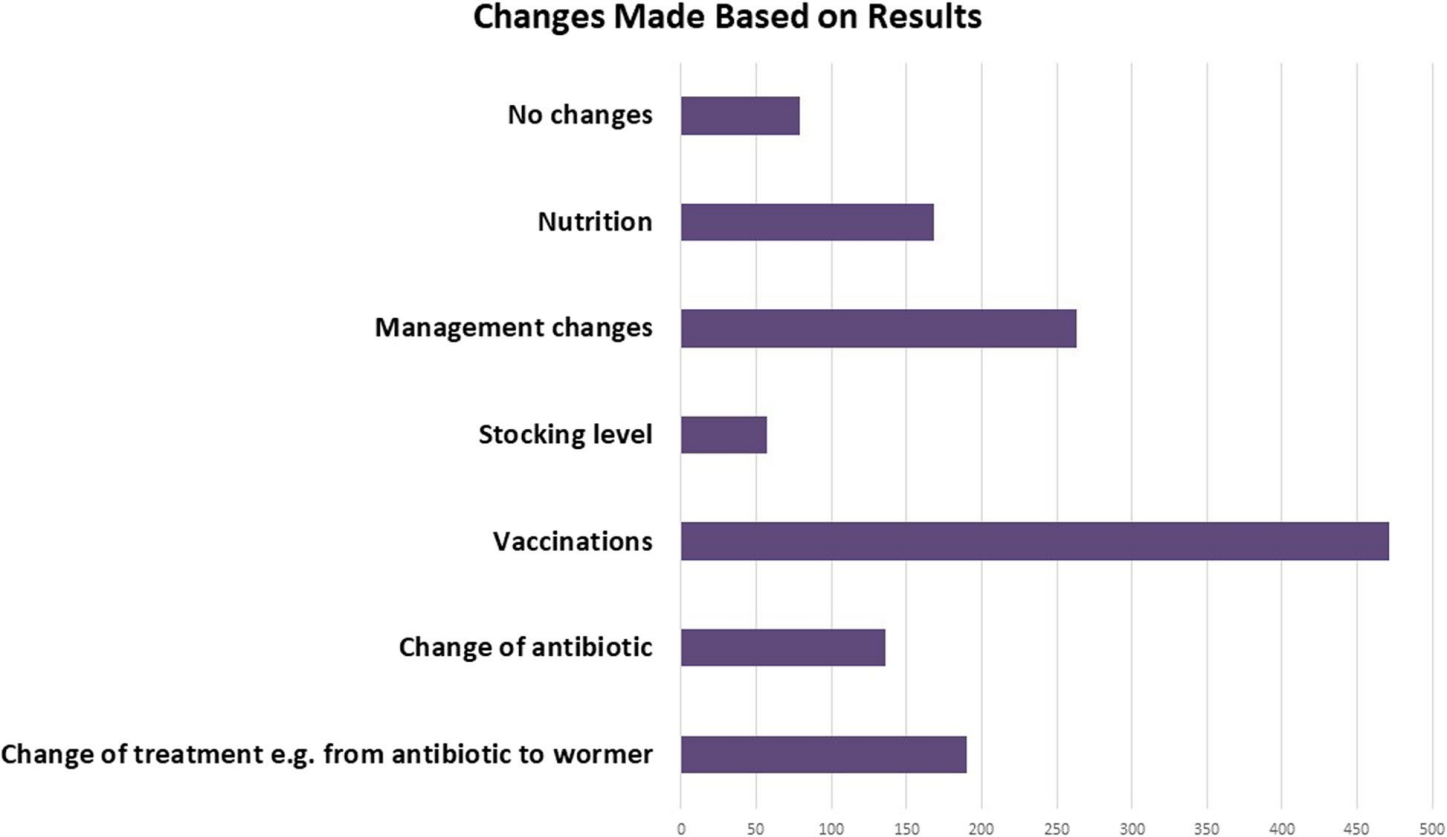
Changes implemented based on results from the RVL

**Fig. 8:**
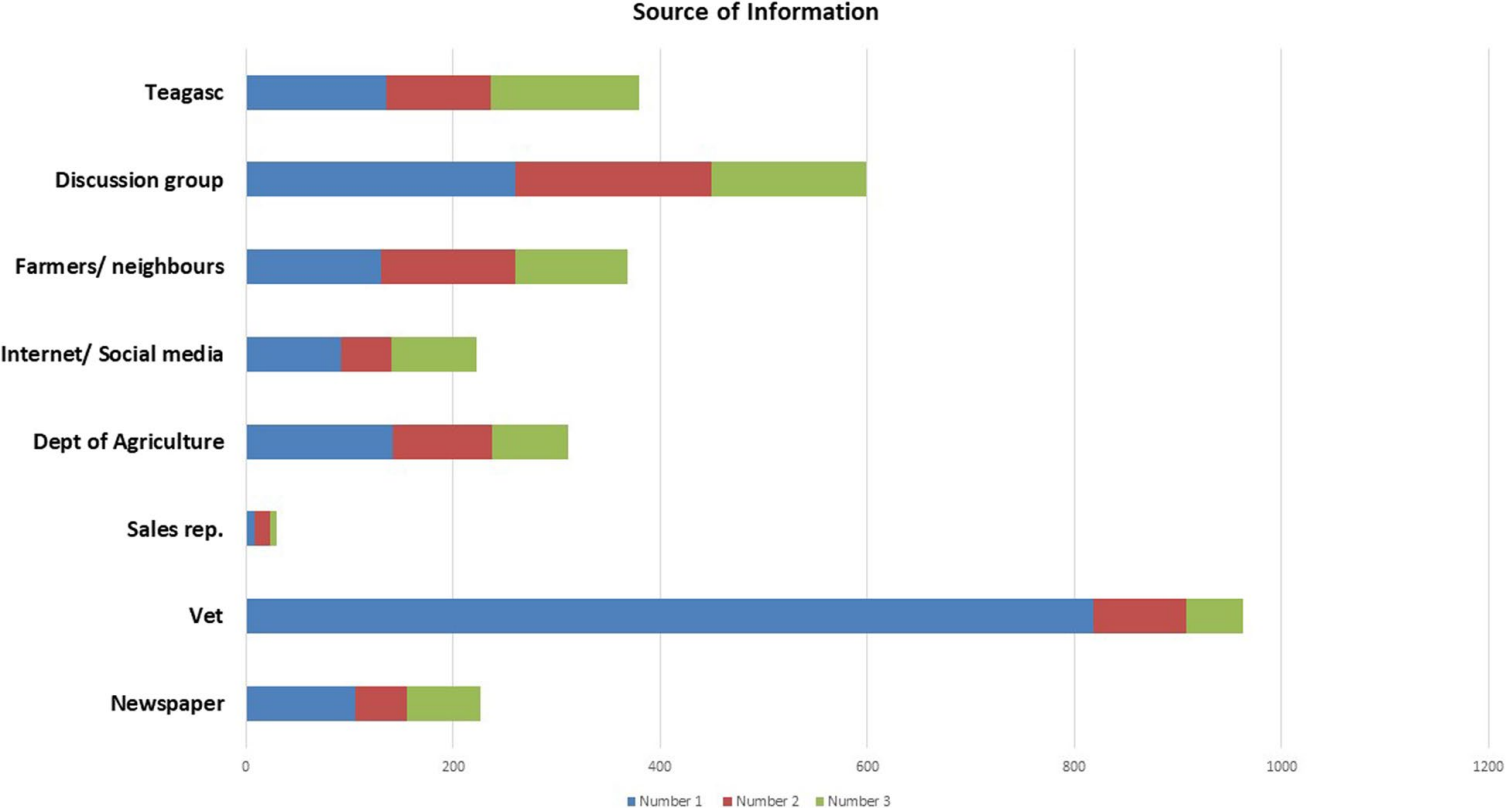
Source of veterinary information

Implementation of vaccination protocols was the main change implemented based on results, followed by management changes and the use of different treatments, e.g. switching from antibiotic to parasite treatment (Fig. 7). Source of information on animal disease was primarily received from vets, followed by discussion groups or Teagasc (Fig. 8). The average number of vet call-outs per year ranged from one to fifty calls.

Significant associations between dependent variables and being identified in the farmers top three reasons to submit/ not to submit are shown in Table 1 and Table 2, including sheep enterprises being more likely than dairy to choose distance and cost as a reason not to submit. Those engaging in some level of dairying were more likely than other enterprise types to submit if they feared a contagious or zoonotic disease. Larger herd size and those who didn’t own the herd were more likely to have vaccination and treatment guidance as a top reason to submit.

**Table 1:** Significant associations between independent and dependant variables (top reasons to submit to the RVL). Independent variables included in the models were enterprise type, herd owner or not, sex, age (categorised into < 40 years, 40–65 years and >  65 years), and above/below median stock number. Respondents were asked to pick their top three reasons why they would submit; however, a number of respondents ranked all answer options from 1 to 12. Therefore answers were categorised into being selected as a top 3 reason or not

Dependent Variable	Odds Ratio	***P*** Value	Conf. Interval (95%)
Independent Variable			
**Reasons to submit to lab**			
**Increased awareness of lab service?**			
Part time vs. Fulltime	0.5	0.023	0.3, 0.9
**Fear of disease that might affect humans**			
Dairy only vs. Beef only	2.0	0.008	1.2, 3.4
Dairy & Beef vs. Beef only	2.4	0.001	1.4, 4.0
**Animals might have contagious disease**			
Dairy only vs. Beef only	2.2	< 0.001	1.5, 3.2
Dairy & Beef vs. Beef only	2.1	< 0.001	1.5, 3.1
Dairy youngstock/ calves only vs. Beef only	3.8	0.017	1.3, 11.6
**To guide treatment / vaccinations**			
Larger stock number vs lower stock number	1.6	< 0.001	1.2, 2.1
Not herd owner vs herd owner	2.2	0.034	1.1, 4.5
**If multiples sick or dead**			
< 40 years vs. > 65 years	2.0	0.007	1.2, 3.4
40–65 years vs. > 65 years	1.6	0.05	1.0, 2.5
Sheep only vs. dairy only	2.7	< 0.001	1.6, 4.6
**Advised by your vet**			
Fulltime vs. Part time	1.5	0.002	1.2, 2.0
**Sudden deaths**			
Beef only vs. Dairy only	1.7	0.014	1.1, 2.5
Sheep only vs. Dairy only	2.4	0.001	1.4, 4.1
**Adult Animal Deaths**			
Mixed vs. Beef only	0.5	0.043	0.3, 1.0

*P* Value: Significant *P* ≤ 0.05

**Table 2:** Significant associations between independent and dependant variables (top reasons to NOT submit to the RVL). Independent variables included in the models were enterprise type, herd owner or not, sex, age (categorised into < 40 years, 40–65 years and >  65 years), and above/below median stock number. Respondents were asked to pick their top three reasons why they would not submit; however, a number of respondents ranked all answer options from 1 to 12. Therefore answers were categorised into being selected as a top 3 reason or not

Dependent Variable	Odds Ratio	***P*** Value	Conf. Interval (95%)
Independent Variable			
**Top Reasons NOT to submit to lab**			
**Not aware of lab service**			
Larger stock number vs. smaller stock number	0.5	0.001	0.3, 0.7
**Lack of time**			
< 40 years vs. > 65 years	2.3	0.001	1.4, 3.7
40–65 years vs. > 65 years	2.1	0.001	1.4, 3.1
**Lack of useful results previously**			
Larger stock number vs. smaller stock number	2.0	< 0.001	1.4, 2.7
**Cost**			
< 40 years vs. > 65 years	2.3	0.026	1.1, 4.6
Beef only vs. Dairy only	3.2	0.001	1.6, 6.3
Sheep Mixed vs. Dairy only	3.0	0.002	1.5, 6.2
Sheep only vs. Dairy only	5.6	< 0.001	2.6, 12.1
**Distance from Lab**			
Sheep mixed vs. Dairy only	2.1	0.001	1.4, 3.1
Sheep only vs. Dairy only	2.7	< 0.001	1.6, 4.6
**I accept a number of losses**			
Dairy only vs. Beef only	1.6	0.035	1.0, 2.6
Dairy and beef vs. Beef only	1.8	0.014	1.1, 2.8
Larger stock number vs. smaller stock number	1.4	0.026	1.0, 1.6
**Vet made diagnosis on farm**			
Dairy only vs. Sheep	2.4	0.002	1.4, 4.2
Beef only vs. sheep only	2.5	0.004	1.3, 3.9
Dairy and beef vs. sheep	2.5	0.001	1.5, 4.3
**Diagnosis made by non-vet**			
> 65 years vs. < 40 years	2.7	0.010	1.3, 5.9

*P* Value: Significant *P* ≤ 0.05

## Discussion

One of the most positive findings from this survey is the desire of participants to submit to the laboratories to guide treatment and vaccination protocols. Concern relating to antimicrobial resistance has increased over the past number of years with the emergence of multi-drug resistant “superbugs”. A number of these infections represent a serious threat to human health. It is estimated that each year, drug-resistant infections result in 25,000 patient deaths in the European Union [8]. There is growing concern regarding the impact of antimicrobial use in agriculture on the emergence of antimicrobial-resistant bacteria. This study highlights the critical role that DAFM RVLs can play in responsible antimicrobial use. The results highlight farmers’ commitment to prioritising herd health and implementing appropriate prevention and treatment strategies. Preventing further outbreaks and guidance related to vaccination and correct treatment options were also noted by McFarland et al., (2020) [5]. As EU restrictions will likely limit drug availability it is likely this guidance will continue to be a major factor in decisions to submit to the laboratories.

A study of Irish farmers by McMahon et al., (2017) [9] found the low level of awareness among farmers of the spread of disease from animals to humans was of concern. Interestingly fear of a zoonotic disease was chosen by a number of participants as a top reason to submit. Those involved in dairying were more likely to choose this as a top reason to submit compared to other enterprises. Given the short timeline for milk to reach the food chain compared to beef and the likely awareness of dairy farmers that specific pathogens can be spread via milk consumption, making pasteurisation advisable, it is possible dairy farmers are more aware of the risk than other enterprises. Although it cannot be insinuated from this study that those involved in other enterprise types are unaware of the zoonotic risk, as suggested by McMahon and Sheehan [9], it may be of benefit to further educate all segments of the farming community about the potential biohazards on farms and the appropriate measures required to mitigate the risk of zoonotic disease, including submission of samples to RVLs to investigate if conditions on farm could potentially pose a zoonotic risk.

To limit the bias of sampling and promote the submission of samples from a diverse population, the PVP, pathologist, and farmer relationship is of great importance in animal health surveillance [2]. Interestingly when the responses of those who had never submitted to the RVL were analysed, the key factor that would prompt them to submit samples in future was if they were advised by their vet, while constraints such as distance and labour will still need to be overcome, results highlight the pivotal role PVPs play in disease surveillance on Irish farms. Gates and Earl [10] have also acknowledged the importance of farmer-veterinarian relationships. Furthermore, the results of this study showed that the main deciding factor in not submitting carcasses was a prior diagnosis made on the farm by the PVP. Positively this may indicate that PVPs prioritise unusual cases or mass mortality that will be of particular value in disease surveillance. This message to prioritise unusual cases will need to be continuously enforced to PVPs to ensure testing capacity is not overwhelmed. It will also be important that animal health authorities ensure that clear protocols and adequate resourcing are in place to manage system submissions, as suggested by Vial and Berezowski [11], especially given results by Limon and Lewis [12] showing that farmers can lose trust if they perceive that the government is not responding to their concerns. Trust in animal health authorities has been noted by Gates and Earl [10] as an essential deciding factor in the notification of disease outbreaks.

Annoyance at inconclusive results was identified in this study and by McFarland et al. [5]. Several factors can influence diagnostic success. Success can be hindered by the type, quality of samples submitted and availability of various diagnostic tests [13]. Clune and Beetson [13] highlighted that understanding the factors that influence the ability of pathologists to reach a diagnosis will allow PVPs to advise clients on the likelihood of investigations yielding a successful diagnosis. It will be important that both PVPs and farmers are educated on factors such as submission of appropriate samples, avoiding chronic cases, opting for fresh carcasses, awareness of various test sensitivity and specificity, importance of submitting a representative number of carcasses and repeat sampling to enhance chances of correct diagnosis. Additionally, investigations relating to perinatal mortality where non-infectious causes are common [14], negative results can often be misinterpreted as being ‘inconclusive’. It is crucial that the value of ‘negative’ results is communicated to the farmers and the importance of the exclusion of major infectious pathogens or zoonotic agents are highlighted. Improvement in communication relating to client expectations and goals may improve satisfaction in investigation outcomes [15]. Furthermore, while the majority of those who submitted samples received results within 2–5 days surprisingly some farmers reported never receiving them. It will be important to enhance communication channels between RVLs, PVPs and farmers to ensure that results are relayed back to the farmers in a timely fashion.

As with McFarland and Macken-Walsh [5] critical reasons identified by participants to not submit to the RVLs included distance and lack of time. McFarland and Macken-Walsh [5] et al. noted that for many dairy farmers, springtime was a period of increased mortality and coinciding with the time of increased workloads, which may influence the decision to submit. However, it was noted in that study that if multiple fatalities began to occur, some farmers would make time to submit irrespective of workload. Given the geographical distribution of many sheep farms in Ireland, including hill flocks, it is perhaps unsurprising that sheep flocks were more likely to pick distance as a key reason not to submit to the RVLs. Proposed carcass collection points (as envisaged for the strategic development of DAFM laboratories) will hopefully lessen the problem of distance from RVLs presented to several respondents. Unusually for some participants, the cost was a factor in deciding to submit. The service provided by the RVLs is highly subsidised, and results from this study suggest that clarification is required to inform farmers that the service is not cost prohibitive. This message especially needs to be communicated to sheep and beef farmers as they were more likely than dairy farmers to identify this as an issue, potentially a refection of the lower margins of these enterprise types.

As with McMahon and Sheehan [9] the vet was the primary source of information on animal diseases, closely followed by discussion groups and Teagasc. Given that larger herd size is a known risk factor for various diseases, e.g. IBR [16, 17], it is perhaps unsurprising larger sized herds were submitting to guide treatment and vaccination protocols, presumably to limit disease transmission. Many of the conditions highlighted in the current study align with the diseases identified most frequently in the All Island disease surveillance reports [18]. Animal Health Ireland is currently running effective control programmes for many of the non-regulated diseases identified, e.g. Cellcheck.^1^ Given the high proportion of respondents who identified lameness as an issue on the farm, however, it may be a condition that requires further research in an Irish context as other studies have identified the need to quantify and address lameness issues on farms [19] . A potential weakness of this paper is survey bias. By distributing the survey at RVLs and discussion groups, responses may not reflect the experiences of the ‘hard to reach farmer’ including the ‘reclusive traditionalist’ as defined by Jansen et al., (2010) [20]. Future work should aim to examine the interactions of such groups with RVLs. To engage with such farmers, it will be important that there are proactive communication strategies tailored to the specific needs of these groups [20] and ensure they are aware of the RVL services available.

## Conclusion

Results show how positive engagement between stakeholders and the RVLs promotes optimal animal health and responsible antimicrobial use, aiding the implementation of farm vaccination strategies. Results also show the critical role PVPs will play in continued disease surveillance on the farm. Enhanced communication between farmers, PVPs and RVLs will be required to ensure optimal samples are submitted to maximise diagnostic success and ensure results are relayed appropriately to farmers to minimise frustration. Maintaining engagement with all farming sectors will be essential in promoting successful animal health surveillance, and overall promoting optimal animal health and welfare.

## Data Availability

Not applicable.

## Abbreviations

DAFM: Department of Agriculture, Food and the Marine
RVL: Regional Veterinary Laboratory
PVP: Private Veterinary Practitioner
PME: Post Mortem Examination

## Footnotes

1 CellCheck is the Irish national mastitis control programme, coordinated and facilitated by Animal Health Ireland. It is developed and delivered in partnership with industry bodies representing farmers, processors, service providers and government.
